# Evidence for ammonium conductance in a mouse thick ascending limb cell line

**DOI:** 10.14814/phy2.13379

**Published:** 2017-08-22

**Authors:** Soojung Lee, Jonathan Park, Jun Ming Li, Kathy Li, Inyeong Choi

**Affiliations:** ^1^ Department of Physiology Emory University School of Medicine Atlanta Georgia

**Keywords:** Ammonium conductance, thick ascending limb, *Xenopus* oocytes

## Abstract

In this study, we examined an ammonium conductance in the mouse thick ascending limb cell line ST‐1. Whole cell patch clamp was performed to measure currents evoked by NH_4_Cl in the presence of BaCl_2_, tetraethylammonium, and BAPTA. Application of 20 mmol/L NH_4_Cl induced an inward current (−272 ± 79 pA,* n* = 9). In current‐voltage (*I*–*V*) relationships, NH_4_Cl application caused the *I*–*V* curve to shift down in an inward direction. The difference in current before and after NH_4_Cl application, which corresponds to the current evoked by NH_4_Cl, was progressively larger at more negative potentials. The reversal potential for NH_4_Cl was +15 mV, higher than the equilibrium potential for chloride, indicating that the current should be due to NH_4_^+^. We then injected ST‐1 poly(A) RNA into *Xenopus* oocytes and performed two‐electrode voltage clamp. NH_4_Cl application in the presence of BaCl_2_ caused the *I*–*V* curve to be steeper. The NH_4_^+^ current was retained at pH 6.4, where endogenous oocyte current was abolished. The NH_4_^+^ current was unaffected by 10 *μ*mol/L amiloride but abolished after incubation in Na^+^‐free media. These results demonstrate that the renal cell line ST‐1 produces an NH_4_^+^ conductance.

## Introduction

${\text{NH}}_{4}^{+}$ is a key buffer component that regulates blood pH. In essence, the kidneys excrete ${\text{NH}}_{4}^{+}$ to urine as they produce ${\text{HCO}}_{3}^{-}$, and the mechanism by which ${\text{NH}}_{4}^{+}$ excretion results in net acid excretion involves a series of sophisticated ${\text{NH}}_{4}^{+}$ transport processes in different parts of the nephron (Weiner and Verlander 2013; Hamm et al. 2015). One of the nephron segments that play key roles in ${\text{NH}}_{4}^{+}$ excretion is the thick ascending limb (TAL) (Mount 2014). ${\text{NH}}_{4}^{+}$ transport in the TAL involves the Na/K/2Cl cotransporter NKCC2 (Good et al. 1984; Kinne et al. 1986), K/NH_4_ exchange and ${\text{NH}}_{4}^{+}$ conductance (Amlal et al. 1994; Attmane‐Elakeb et al. 2001) in the luminal membrane of the tubule, and the Na/H exchanger NHE4 (Bourgeois et al. 2010) in the basolateral membrane. The basolateral ${\text{NH}}_{4}^{+}$ transport is also mediated by a dissociation of intracellular ${\text{NH}}_{4}^{+}$ into NH_3_ and H^+^ and subsequent NH_3_ exit to the interstitium.

In the luminal membrane of the TAL, NKCC2 is the major fraction of the active ${\text{NH}}_{4}^{+}$ flux. Nonetheless, in vitro studies reveal that K/NH_4_ exchange and ${\text{NH}}_{4}^{+}$ conductance can contribute to the ${\text{NH}}_{4}^{+}$ transport by 35–50% (Amlal et al. 1994; Attmane‐Elakeb et al. 2001). K/NH_4_ exchange is barium‐ and verapamil‐sensitive, whereas ${\text{NH}}_{4}^{+}$ conductance is barium‐insensitive and amiloride‐sensitive (Amlal et al. 1994). The two pathways exhibit biophysical and pharmacological characteristics that distinguish them from other ${\text{NH}}_{4}^{+}$‐transporting proteins. Despite such physiological and functional significance, our understanding of these pathways is limited because their molecular entities are presently unknown.

In this study, we examined an ${\text{NH}}_{4}^{+}$ conductance in the mouse TAL cell line ST‐1. This cell line is nonpolarized and exhibits many features characteristic of TAL cells (Kone et al. 1995; Kone and Higham 1999; Lee et al. 2010). We performed whole cell patch clamp of the cells to identify the ${\text{NH}}_{4}^{+}$ conductance and determine basic electrophysiological properties such as the amount of current, direction, current‐voltage relationship, and reversal potential. We then isolated ST‐1 poly(A) RNA and injected it into *Xenopus* oocytes and performed two‐electrode voltage clamp in an effort to identify a protein conducting ${\text{NH}}_{4}^{+}$. While our search for the protein is in progress, here we report that the ${\text{NH}}_{4}^{+}$ conductance in ST‐1 cells is not identical to the previously reported Cl^–^‐dependent ${\text{NH}}_{4}^{+}$ conductance in the TAL of the nephron.

## Methods

### Ethical approval

All experiments in this study were conducted under the National Institutes of Health guidelines for research, and experimental protocols were approved by the Institutional Animal Care and Use Committee at Emory University.

### Cell culture

ST‐1 is a cell line derived from mouse medullary TAL tubules, developed by Bruce Kone (Kone et al. 1995; Kone and Higham 1999). Cell authentication is based on the report (Haas and Hebert 1992) on the expression of bumetanide‐sensitive proteins and our previous report (Lee et al. 2010) demonstrating the expression of the electroneutral Na/HCO_3_ transporter NBCn1 and Cl/HCO_3_ exchanger AE2 in this cell line by immunoblot. Cells were cultured in Dulbecco's modified eagle's medium supplemented with 10% fetal bovine serum, 50 U/mL penicillin and 50 *μ*g/mL streptomycin in a 5% CO_2_ air equilibrated 37°C incubator. For patch clamp recording, cells were seeded on poly‐D‐lysine‐coated coverslips at a density of 8 × 10^4^ cells per well in a 12‐well plate. Recordings were done 2 days later.

### Whole cell patch clamp of ST‐1 cells

Whole cell recording was performed using the protocol by Hayashi et al. (1992) with slight modification. A coverslip on which cells were grown was mounted on a recording chamber affixed on the stage of a Nikon Eclipse T1 inverted microscope, equipped with a MP‐225 motorized micromanipulator. Cells were perfused with solution containing (in mmol/L) 116 NaCl, 6 KCl, 2.4 CaCl_2_, 6 glucose, 10 HEPES, 10 tetraethylamonium (TEA), and 1 BaCl_2_, pH 7.4. The pipette had 116 KCl, 1.2 MgCl_2_, 6 glucose, 10 HEPES, 10 TEA, 10 BAPTA, and 0.01 BaCl_2_ (pH 7.2). Recording was made using an Axopatch 200B amplifier (Molecular Devices, Sunnyvale). Currents were recorded with 20 mmol/L NH_4_Cl, at a holding voltage of ‐70 mV. For amiloride experiments, currents induced by NH_4_Cl were recorded in the absence and then presence of 1 *μ*mol/L amiloride (Sigma‐Aldrich; Cat#: A7410). *I*–*V* relationship was determined by a staircase voltage command between −80 to +15 mV (170 msec duration). Currents were recorded using pClamp 8.0 (Molecular Devices) and signals were low‐pass filtered (3 db at 2 kHz, 8‐pole Bessel filter). Experiments were performed at room temperature.

### Two‐electrode voltage clamp of oocytes

ST‐1 cells grown in 100 mm plates were collected and lysed for poly(A) RNA isolation using RNeasy Mini kit (Qiagen, Germantown). *Xenopus laevis* oocytes at stages V and VI were purchased from Ecocyte Bioscience (Austin). Poly(A) RNA was injected into oocytes (2.7 ng per oocyte in 46 nl) and controls were water only. Oocytes were maintained for 3 days at 18°C before use. A RC‐24N recording chamber (Warner Instrument, Hamden) was filled with perfusion solution containing (in mmol/L) 20 LiCl, 80 NaCl, 3.8 BaCl_2_, and 5 HEPES, pH 7.4. For acidic solution, the pH was adjusted to 6.4. An oocyte was placed in the chamber and impaled with two glass electrodes filled with 3 mol/L KCl (a tip resistance of 0.5–2 mol/LΩ). The oocyte was clamped at –60 mV using a OC‐725C voltage‐clamp amplifier (Warner Instrument). NH_4_Cl solutions were made by replacing LiCl at equimolar concentrations. Na^+^‐free solutions were made by replacing Na^+^ with N‐methyl‐D‐glucamin (NMDG). For drug sensitivity experiments, 10 *μ*mol/L amiloride and 200 *μ*mol/L bumetanide (Sigma‐Aldrich; Cat #: B3023) were used. The voltage command was from –140 to +40 mV with 20 mV increments (100 msec duration). The voltage command after NH_4_Cl application was made when the current reached steady state (~4 min) (Lee and Choi 2011). Voltage signals were sampled by a Digidata 1322A (Molecular Devices) and data were acquired using pClamp 8.0.

### Statistical analysis

Data were reported as mean ± standard error. The level of significance was determined using (1) paired, two‐tailed Student *t*‐test for comparison of slopes before and after ${\text{NH}}_{4}^{+}$ application to oocytes or ST‐ cells, and comparison of the currents before and after NH_4_Cl application in patch clamp; (2) unpaired, two‐tailed Student *t*‐test for comparison of *G*_NH4_ between RNA‐injected versus water‐injected oocytes; and (3) two‐way ANOVA with Bonferroni post hoc test for comparison of slopes in the presence of bumetanide and in Na^+^‐free solutions between different groups of oocytes. The *P* value of less than 0.05 was considered significant. Analysis was made using Microsoft Office Excel add‐in program Analysis ToolPak (Redmond, WA).

## Results

### ${\text{NH}}_{4}^{+}$ conductance in ST‐1 cells

**Figure 1:**
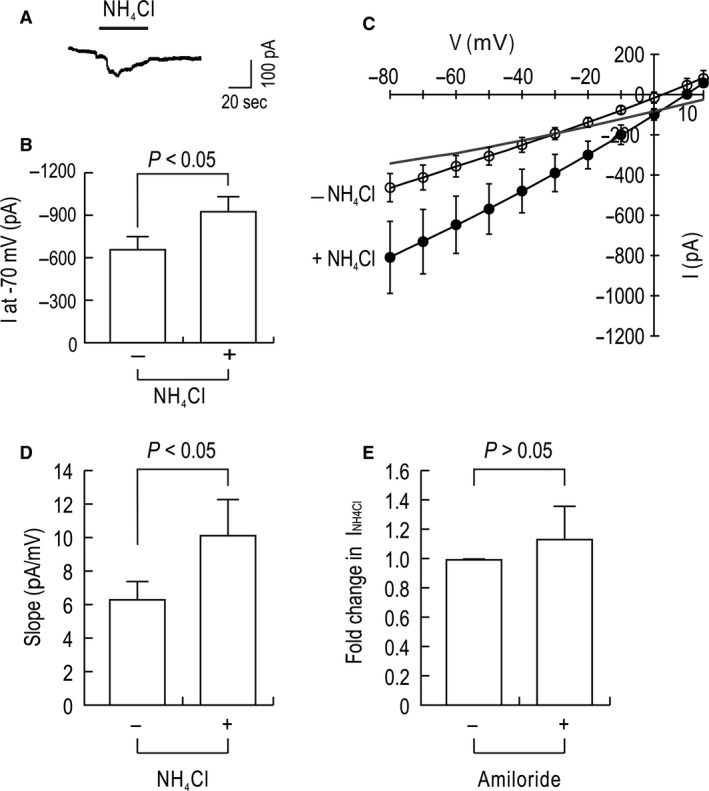
NH_4_^+^ currents in ST‐1 cells measured by whole cell patch clamp. (A) An example of a whole cell current evoked by 20 mmol/L NH_4_Cl in an ST‐1 cell. The recording was performed in the presence of 2 mmol/L BaCl_2_, 10 mmol/L TEA, 5 mmol/L BAPTA. The holding potential was −70 mV. NH_4_Cl replaced LiCl at the equimolar concentration. (B) Mean peak current. The difference in currents before and after NH_4_Cl application represents an NH_4_Cl‐mediated current (*n* = 9). (C) *I*–*V* relationships. Peak or steady‐state currents at different voltages were acquired by the voltage command stepping from −80 to +15 mV (*n* = 5). The difference between the two *I*–*V* curves is shown in a gray line. (D) Mean slope determined from the *I*–*V* curves. (E) Effect of 1 *μ*mol/L amiloride on NH_4_Cl‐induced currents (*I*_NH__4Cl_). Data are presented as fold change relative to I_NH__4Cl_ produced without amiloride (*n* = 6 for each).

We performed patch clamp recording of ST‐1 cells in a whole cell configuration to assess ${\text{NH}}_{4}^{+}$ conductance. Recordings were performed in the presence of 1 mmol/L BaCl_2_ and 10 mmol/L TEA to block K channels, and 5 mmol/L BAPTA to block intracellular Ca^2+^ increase. Figure 1A shows an example of the inward current evoked by 20 mmol/L NH_4_Cl at the holding potential of −70 mV. As in many cells, the current was decayed slowly but we also frequently observed steady‐state currents after reaching a peak. Figure 1B shows the currents measured before and after NH_4_Cl application. The mean difference between the two values, corresponding the current evoked by NH_4_Cl, was −272 ± 79 pA (*P* < 0.05; *n* = 9). In *I*–*V* relationships (Fig. 1C), NH_4_Cl application caused the *I*–*V* curve to shift down in an inward direction. The difference in the two curves, corresponding to the currents evoked by NH_4_Cl at different voltages, was progressively larger at more negative potentials. The reversal potential for NH_4_Cl was +15 mV, higher than the equilibrium potential for chloride (−4 mV) estimated by chloride concentrations in the patch pipette and bath solutions. Thus, the inward current evoked by NH_4_Cl is not due to Cl^–^, but to ${\text{NH}}_{4}^{+}$. The ${\text{NH}}_{4}^{+}$ conductance (*G*_NH4_) determined by the difference in the slopes of the two *I*–*V* curves was 4.4 nS (Fig. 1D). In other experiments, we determined the effect of amiloride on the currents (Fig. 1E). Measured at the holding potential of −70 mV, the currents were unaffected by 1 *μ*mol/L amiloride (*P* > 0.05; *n* = 6).

### *G*_NH4_ induced by ST‐1 proteins

**Figure 2:**
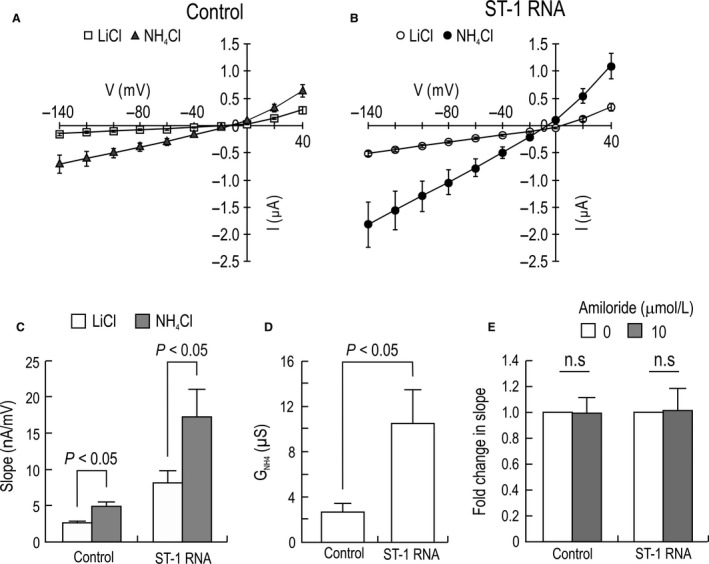
NH_4_^+^ conductance in oocytes injected with water or poly(A) RNA of ST‐1 cells. (A and B) *I*–*V* relationships of NH_4_^+^ currents measured by two‐electrode voltage clamp. Steady‐state currents were recorded before and after switching solutions from 20 mmol/L LiCl to the equimolar concentration of NH_4_Cl (*n* = 7 RNA‐injected oocytes and 5 water‐injected controls). (C) Mean slope of the *I*–*V* curve. Slope was determined near zero‐current voltage. (D) NH_4_^+^ conductance (*G*_NH__4_). *G*_NH__4_ represents the difference between the two slopes before and after NH_4_Cl application. (E) Effect of 10 *μ*mol/L amiloride on the NH_4_Cl‐induced slope. Data are presented as fold change relative to the slope before amiloride treatment (*n* = 6 for each).

To express ST‐1 proteins in *Xenopus* oocytes, we isolated poly(A) RNA from the cells and injected it into oocytes. Figure 2A shows *I*–*V* relationships of water‐injected control oocytes, obtained before and after application of 20 mmol/L NH_4_Cl in the presence of 3.8 mmol/L BaCl_2_. NH_4_Cl caused the *I*–*V* curve to shift down in an inward direction. This shift is due to endogenous oocyte conductance (Lee and Choi 2011). A slight increase in an outward current was observed, probably due to LiCl that depolarizes oocyte membranes. In oocytes injected with poly(A) RNA (Fig. 2B), the basal current before NH_4_Cl application was higher, due to Na^+^ as describe below. NH_4_Cl caused the *I*–*V* curve to shift down with a steeper slope. At −60 mV, the inward current in these oocytes was −780 ± 164 nA, significantly higher than −240 ± 12 nA in controls. The reversal potential (the voltage where the two *I*–*V* curves intersect) was similar between control oocytes and RNA‐injected oocytes, indicating that the currents are likely produced by nonselective cation channels. Figure 2C shows the comparison of the slopes determined near the reversal potential. RNA‐injected oocytes had a more increased slope in response to NH_4_Cl (*P* < 0.05 for both; *n* = 7 for RNA‐injected oocytes and 5 for water‐injected controls). Thus, the *G*_NH4_ in RNA‐injected oocytes was significantly larger than that of controls (*P* < 0.05; Fig. 2D). In other experiments, we examined the effect of amiloride on the slope induced by NH_4_Cl (Fig. 2E). We found no significant change by 10 *μ*mol/L amiloride (*P* > 0.05; *n* = 5 for each).

### Retention of ST‐1 *G*_NH4_ at acidic pH

**Figure 3:**
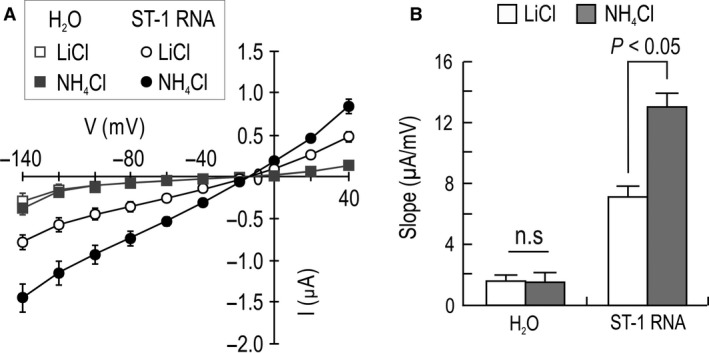
NH_4_^+^ conductance at pH 6.4. (A) *I–V* relationships recorded at pH 6.4. Recordings were made using the protocol described in Figure 2. Data are from 5 RNA‐injected oocytes and 5 controls). (B) Mean slope of the *I*–*V* curve. Slopes were determined using the *I*–*V* relationships in *A*.

We performed *I*–*V* recordings in solutions with pH 6.4 to determine whether endogenous oocyte conductance was responsible for increased G_NH4_ in RNA‐injected oocytes. Endogenous oocyte ${\text{NH}}_{4}^{+}$ transport is known to be inhibited at low pH (Nakhoul et al. 2010). We found that while the *G*_NH4_ in control oocytes was abolished at pH 6.4, the one in RNA‐injected oocytes was still induced under the same condition (*P* < 0.05; Fig. 3). Thus, the *G*_NH4_ in RNA‐injected oocytes is mainly induced by heterologously expressed ST‐1 proteins.

### Inhibition of *G*_NH4_ by incubation in Na^+^‐free solutions

**Figure 4:**
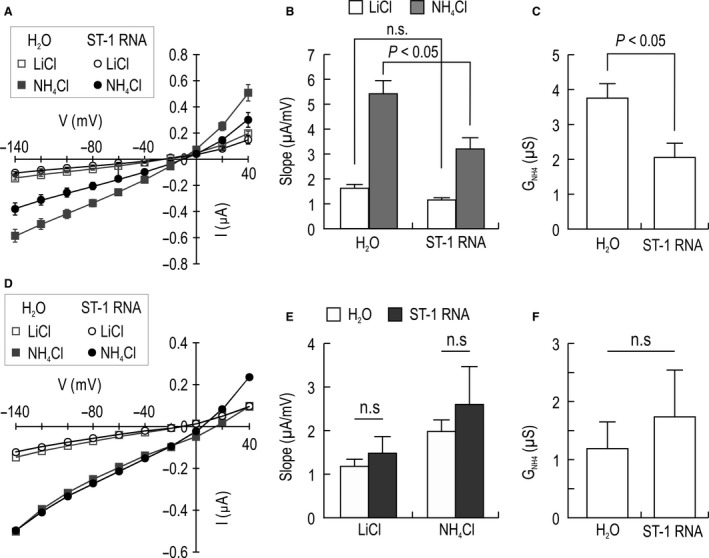
Inhibition of *G*_NH__4_ in Na^+^‐free media. (A) *I*–*V* relationships of NH_4_^+^ currents in Na^+^‐free solutions (NMDG replaced Na). Oocytes were incubated in Na^+^‐free bath ND96 solution for >3 h (*n* = 7 RNA‐injected oocytes and 6 controls). *I‐V* relationships were determined under Na^+^‐free conditions. (B) Mean slope. (C) *G*_NH__4_. (D) Representative *I*–*V* relationships of oocytes treated with 200 *μ*mol/L bumetanide in Na^+^‐free solutions. (E) Comparison of mean slopes between RNA‐injected oocytes and controls treated with bumetanide (*n* = 6 RNA‐injected oocytes and 7 controls). (F) *G*_NH__4_. n.s: not significant.

To test whether the *G*_NH4_ in RNA‐injected oocytes is affected by Na^+^, we incubated oocytes in Na^+^‐free solutions (NMDG replaced Na^+^) and determined *I*–*V* relationships. The incubation time was at least 3 h to ensure that intracellular Na^+^ is substantially low to minimize Na^+^ efflux. Figure 4A shows the *I*–*V* relationships under Na^+^‐free conditions. NH_4_Cl application caused the *I*–*V* curve of control oocytes to inwardly shift down, indicating negligible effect of Na^+^ removal on endogenous conductance. In contrast, Na^+^ removal induced two changes in RNA‐injected oocytes. First, the *I*–*V* curve before NH_4_Cl application was similar to the control curve. Second, the shift in the curve after NH_4_Cl application was smaller than the control. These changes were evident when the slopes of the curves were compared (Fig. 4B). Compared to controls, RNA‐injected oocytes had a similar slope of the basal current (*P* > 0.05; *n* = 7 for RNA‐injected oocytes and 6 for controls) but a small slope of the ${\text{NH}}_{4}^{+}$ current (*P* < 0.05). This resulted in a smaller *G*_NH4_ in RNA‐injected oocytes (Fig. 4C). To test whether the ‘below‐control’ decrease in *G*_NH4_ is associated with NKCC2, we treated oocytes with 200 *μ*mol/L bumetanide under Na^+^‐free conditions. Figure 4D shows an example of *I*–*V* relationships after treating with bumetanide. The *I*–*V* curves after NH_4_Cl application were nearly superimposed between controls and RNA‐injected oocytes, indicating that bumetanide unleashed the excessive inhibition of the *G*_NH4_ by Na^+^ removal. The two groups of oocytes had similar slopes and *G*_NH4_ (*P* < 0.05; *n* = 6 for RNA‐injected oocytes and 7 for controls), as demonstrated in Figure 4E and F. We also used 500 *μ*mol/L of bumetanide and found the same effects (data not shown).

## Discussion

The aim of this study was to obtain the basic electrophysiological properties of the ${\text{NH}}_{4}^{+}$ conductance in the TAL cell line ST‐1 for future study of its molecular identification. Using a homogeneous cell line and its protein expression in a relatively simple heterologous oocyte system, we identified an ${\text{NH}}_{4}^{+}$ conductance and obtained information on the direction of the current flow, amounts of currents produced, *I*–*V* relationships, and ion dependence. An interesting finding is that this conductance is not inhibited by amiloride and appears to be dependent upon Na^+^. This finding is significant as it provides evidence that the ${\text{NH}}_{4}^{+}$ conductance in ST‐1 is different from the previously reported Cl^–^‐dependent ${\text{NH}}_{4}^{+}$ conductance in the TAL of a nephron.

Amlal et al. (1994) have reported the ${\text{NH}}_{4}^{+}$ conductance in the isolated rat medullary TAL by monitoring membrane depolarization of the tubules using DiSC_3_(5). While this approach determines whether there is a current flowing across the membrane, it does not measure actual membrane conductance. Current is proportional to conductance at a constant voltage, and a voltage or current clamp should be done to correctly measure a membrane conductance. In our study, we directly measured the current evoked by NH_4_Cl. The current was inwardly directed with a positive reversal potential and a positive *G*_NH4_ was observed. These data are consistent with an inward movement of positively charged ${\text{NH}}_{4}^{+}$ ions. This inward current is unlikely mediated by Cl^–^. Given the Cl^–^ concentrations in the patch pipette and in the bath, the equilibrium potential for Cl^–^ is estimated to be slightly negative. The voltage‐clamp experiments of oocytes shows that the ${\text{NH}}_{4}^{+}$ conductance is produced in the presence of barium. Patch clamp of ST‐1 cells also shows ${\text{NH}}_{4}^{+}$ conductance in the presence of barium and TEA, consistent with the report that ${\text{NH}}_{4}^{+}$ is poorly transported by K channels in the TAL (Attmane‐Elakeb et al. 2001).

We found that the *G*_NH4_ was inhibited after incubation in Na^+^‐free solutions (Fig. 4). This Na^+^‐dependent inhibition is unexpected because an ${\text{NH}}_{4}^{+}$ current is considered to occur via K^+^ channels (or transporters) by replacing a K binding site. One explanation is that Na^+^‐free incubation has lowered intracellular pH by reversing the Na/H exchangers and then inhibited the *G*_NH4_. However, this is unlikely to happen because the *G*_NH4_ can be produced at acidic pH as shown in Figure 3. Another explanation is that Na^+^‐free solutions has inhibited Na^+^ channels or Na^+^ channel‐like proteins, which may induce ${\text{NH}}_{4}^{+}$ currents. ${\text{NH}}_{4}^{+}$ affects activities of epithelial sodium channel ENaC (Nakhoul et al. 2001) and gates acid‐sensing ion channel ASICs (Pidoplichko and Dani 2006). Nonetheless, we do not think that ENaC and ASICs are responsible for the *G*_NH4_ in ST‐1 because these two proteins are very sensitive to amiloride (Benos 1982; Wemmie et al. 2006). We found no amiloride sensitivity of the *G*_NH4_. The $NH_{4}^{+}$ conductance in the TAL is sensitive to amiloride given that 1 *μ*mol/L amiloride completely abolishes the NH_4_Cl‐induced membrane depolarization in the isolated TAL tubules (Amlal et al. 1994). The TAL does not express ENaC although we note that ENaC antibodies detect signals in the luminal side of rat TAL (Brown et al. 1989). Taken together, we think that the molecule responsible for ${\text{NH}}_{4}^{+}$ conductance in ST‐1 is a novel protein that is not inhibited by amiloride and has sensitivity to Na^+^, probably to intracellular Na^+^. ST‐1 RNA‐injected oocytes do not regain *G*_NH4_ after incubation in Na^+^‐free solutions (S. Lee, unpublished observation), implying that the intracellular Na^+^ levels are critical for *G*_NH4_.

What would be a potential role of the *G*_NH4_ in renal ammonium excretion? We think that the conductance would contribute to renal adoptive process in acid‐base disorders. For example, in rats and humans, K^+^ depletion is associated with increased urinary ${\text{NH}}_{4}^{+}$ production and excretion, ultimately developing metabolic alkalosis (Jones et al. 1982; Abu Hossain et al. 2011). While this development is probably due to increased ammoniagenesis in the proximal tubules, K^+^ depletion downregulates NKCC2 in the TAL (Amlal et al. 1998). Thus, it is possible that other mechanisms such as ${\text{NH}}_{4}^{+}$ conductance are upregulated during K^+^ depletion. In addition, the *G*_NH4_ may serve as a fine‐tuning regulator of ${\text{NH}}_{4}^{+}$ transport in the TAL, where luminal ${\text{NH}}_{4}^{+}$ transport is mainly mediated by electroneutral NKCC2. The regulation of *G*_NH4_ by Na^+^ might be a novel mechanism that links NKCC2 to ${\text{NH}}_{4}^{+}$ absorption and subsequent excretion.

In conclusion, our findings are interesting and provide a foundation for future studies of electrophysiological and pharmacological properties of the ${\text{NH}}_{4}^{+}$ conductance. Those data will subsequently lead to obtaining molecular information on an ammonium channel.

## Conflict of Interest

None declared.
